# A novel splicing mutation in the *PKD1* gene causes autosomal dominant polycystic kidney disease in a Chinese family: a case report

**DOI:** 10.1186/s12881-018-0706-6

**Published:** 2018-11-13

**Authors:** Peiwen Xu, Sexing Huang, Jie Li, Yang Zou, Ming Gao, Ranran Kang, Junhao Yan, Xuan Gao, Yuan Gao

**Affiliations:** 1 Center for Reproductive Medicine, Shandong University, Jinan, 250001 China; 2 National Research Center for Assisted Reproductive Technology and Reproductive Genetics, Jinan, 250001 China; 3 The Key laboratory for Reproductive Endocrinology of Ministry of Education, Jinan, 250001 China

**Keywords:** Autosomal dominant polycystic kidney disease, *PKD*1 gene, Novel splice mutation, Frameshift mutation

## Abstract

**Background:**

Autosomal dominant polycystic kidney disease (ADPKD) is the most common monogenic renal disorder in humans, affecting 1 in 400 to 1000 individuals. Mutations *PKD1* (which accounts for 85% of ADPKD and produces polycystin-1) and *PKD2* (produces polycystin-2) are responsible for this disease. These two polycystins are critical for maintaining normal renal tubular structures during kidney development.

**Case presentation:**

We performed genetic analysis on a family with ADPKD. DNA samples extracted from ADPKD patient blood were subject to targeted Next generation sequencing for human a panel of renal disease-related genes. A splicing mutation, c.2854-3C > G (also known as IVS11–3C > G), in the *PKD*1 gene was found in the 3 patients from the family, but was not found in four unaffected relatives and 100 normal control samples. Reverse transcription-PCR (RT-PCR) was performed to analyse the relative mRNA expression in the patient samples. mRNA sequencing showed that 29 bases inserted into the 3′-end of exon 11 in the *PKD*1 gene lead to a frameshift mutation.

**Conclusions:**

The *PKD1* c.2854-3C > G mutation leads to a frameshift mutation during translation of the polycystin-1 protein, which eventually led to ADPKD in the Chinese family.

## Background

Autosomal dominant polycystic kidney disease (ADPKD) is a common hereditary kidney disease characterized by the progressive development and enlargement of renal cysts, typically leading to end-stage renal disease (ESRD) by late middle age [1]. This slowly progressive systemic disease is not only associated with the presence of multiple kidney cysts but also causes multiple manifestations, such as liver cysts and cerebral aneurysms [2].

Most ADPKD are genetically heterogeneous, with the following two identified causative genes: polycystic kidney disease 1 (*PKD*1) (OMIM 601313; 16p13.3), responsible for 85% of patients, and polycystic kidney disease 2 (*PKD*2) (OMIM 173910; 4q22.1), responsible for 15% of patients [3–5].

The *PKD*1 gene is composed of 46 exons spanning ~ 50 kb of genomic DNA. The transcript is 14,136 bp. The predicted polycystin-1 (PC-1) protein consists of 4,302 amino acids (amino acids, aa) [6, 7]. The *PKD*2 gene encodes an ~ 5.0 kb mRNA, which is derived from 15 exons and covers ~ 70 kb of genomic DNA. The predicted polycystin-2 (PC-2) protein which consists of 968 aa, acts as a transient receptor potential (TRP) ion channel involved in the regulation of intracellular Ca2^+^ concentrations [8]. Both proteins interact with each other in kidney primary cilia to form a complex that hypothetically functions as a flow-dependent mechanosensor to promote tubular epithelial cell adhesion, proliferation and differentiation [9, 10]. In total 1,448 different types of mutations have been reported in the Human Gene Mutation Database (HGMD), and the clinical significance of some variations remains unknown.

We discovered a novel pathogenic mutation in the *PKD1* gene, and its underlying aetiology in a family with autosomal dominant polycystic kidney disease.

## Case presentation

### Patients

**Fig. 1:**
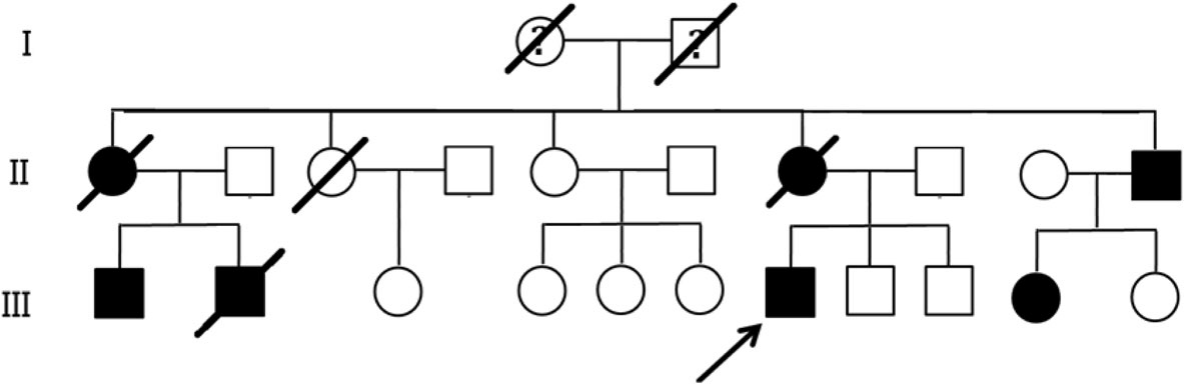
Pedigree of the Chinese ADPKD family. Affected family members are denoted in black. The arrow indicates the proband

We investigated an ADPKD family with seven individuals (Fig. 1). The three affected individuals were 43 years old (Patient II:10), 39 years old (Patient III:1), and 29 years old (Patient III:7) at the time of the investigation. Participants II5, II8, III8 and III9 did not show any polycystic kidney disease. All participants signed informed consent. The experimental protocol was approved by the Institutional Review Board.

**Fig. 2:**
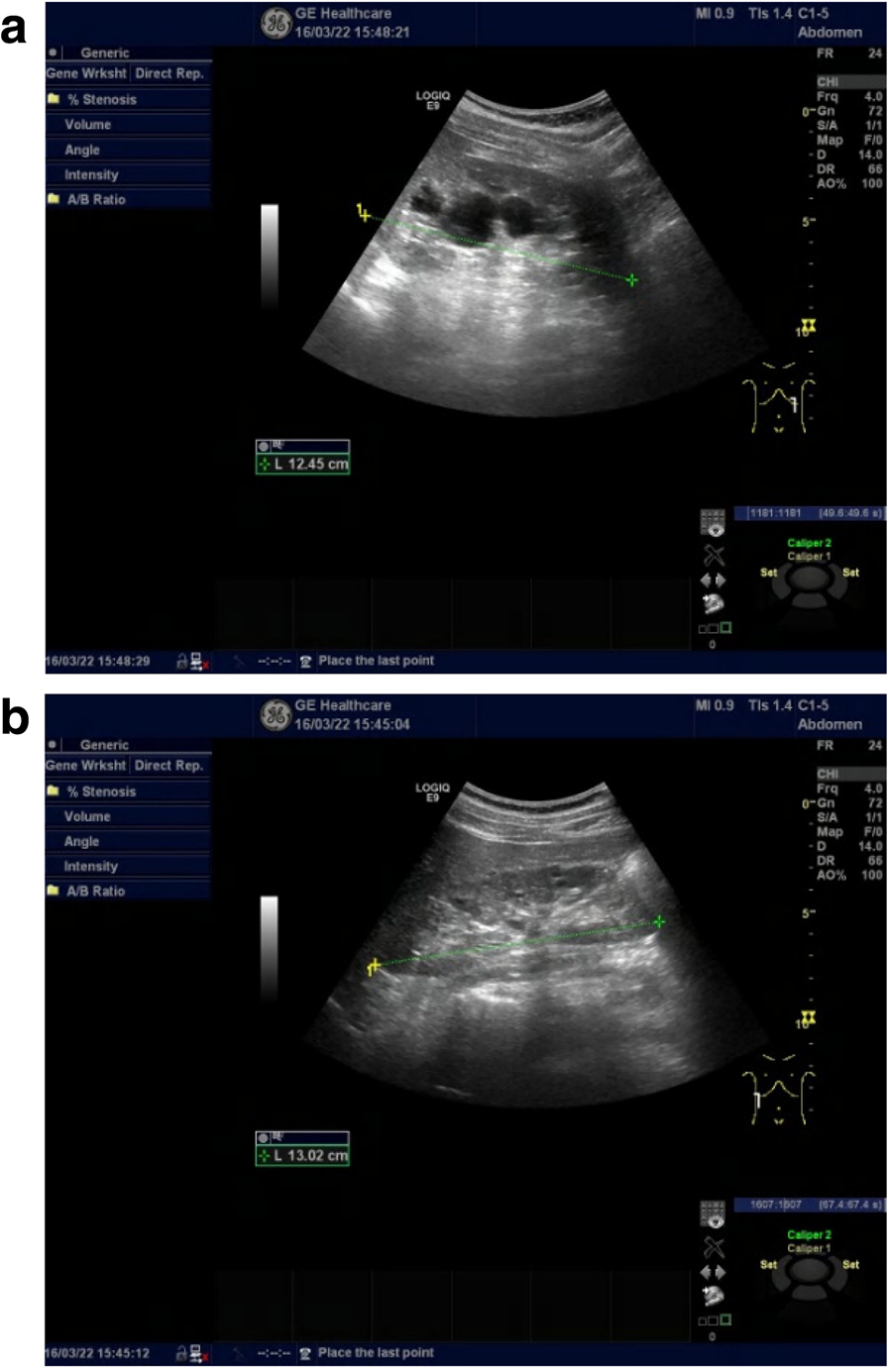
The bilateral renal cysts from the proband III7: (**a**) Left kidney and (**b**) right kidney. At 29 years of age, the left kidney measured 12.5 × 6.5 × 6.7 cm and the thickness of the parenchyma was 1.5 cm. The right kidney measured 13.0 × 4.4 × 7.0 cm and the thickness of the parenchyma was 1.7 cm. Vesicle-like echoes of multiple sizes were observed in both sides of the renal parenchyma. The large size measured 3.1 × 2.8 cm in the left kidney and the large size measured 1.5 × 1.3 cm in the right kidney. No hydronephrosis and good corticomedullary differentiation were observed

The proband (III:7, Fig. 1) is 31 years old(born in 1987), male, 173 cm, 70 kg, and Han Chinese. He has a normal daily routine, eats regularly, does not drinks but smoke. He is self-employed and does not perform physical labour with strong intensity. He has suffered from back pain after manual labour and mild hypertension in recent years. Ultrasound examination data from the proband (III:7) showed a polycystic kidney (initial stage) at 29 years of age (Fig. 2).

II7 is a female with a history of hypertension and had a polycystic kidney detected by ultrasound examination at 42 years of age. She had right polycystic kidney surgery at 43 years of age and died of a polycystic kidney complicated with epilepsy at 50 years of age.

II10 is a male with refractory hypertension and no alcoholism and suffers from back pain. He had a polycystic kidney detected by an ultrasound examination at 35 years of age. He has had no polycystic kidney-related surgeries.

III1 is a male who had a polycystic kidney detected by an ultrasound examination at 36 years of age and a CT scan at 37 years of age. He has a normal daily routine and diet and does not drink. He has had no polycystic kidney-related surgeries.

III2 had a history of alcoholism and polycystic kidney disease and died of a brainstem haemorrhage due to hypertension at 37 years of age. This individual had no polycystic kidney-related surgeries.

II5, II8, III8 and III9 display physical fitness and ultrasound examinations performed on these individuals did not detect a polycystic kidney.

### Genetic analysis

Five- millilitre blood samples were collected from each subject for genomic DNA extraction using the CWBIO Blood Genomic DNA Mini Kit (CWBIO, Beijing, China). The isolated genomic DNA from the proband was then fragmented to 150–200 bp and subjected to DNA library preparation using established Illumina paired-end protocols. Adaptor-ligated libraries were amplified via PCR. *PKD1* and *PKD2* genes capture were performed with the TruSight One Sequencing Panel (Illumina Inc.,USA). The exon-enriched libraries of *PKD1* and *PKD2* genes were sequenced with the Illumina MiSeq platform (Illumina, Inc., USA) according to the manufacturer’s instructions. The sample was sequenced per lane to obtain an average theoretical depth of 106 × . All reads were aligned to the reference human genome (UCSC hg19) with Burrows-Wheeler Aligner (BWA)(v.0.5.9). Local realignment and base quality recalibration of the Burrows-Wheeler aligned reads were then performed using the GATK IndelRealigner(v3.5) and GATK BaseRecalibrator (v3.5), respectively. Single nucleotide variants (SNVs) and small indels were identified by the GATK UnifiedGenotyper(v 3.5). Variants, including SNVs and small indels, were annotated with Annovar (v 2016Feb01). Suspicious candidate variants were obtained by a screening process that combined NGS data with clinical data and prediction results from bioinformatics software (PolyPhen 2, LRT, Mutation Taster, etc.).

We found a heterozygous splice mutation in intron 11 of the *PKD1* gene(NM_001009944.2, c.2854-3C > G, also as IVS11–3 C > G) in the proband. Sanger sequencing was performed to validate the identified variation known to causing ADPKD. Genotyping of c.2854–3 was conducted using an automated ABI3730 DNA sequencer with the 5’-GCTGGAGAGCCGGACAGTG-3′(forward) and 5’-TTCATCCGCTCCACGGTTAC-3′(reverse) primers. Sequencing results were analysed with the Chromas software (v 2.22) and NCBI BLAST tools. The c.2854-3C > G (also as IVS11–3C > G) variant in the *PKD*1 gene was found in the 3 patients from the family, but not in the four unaffected relatives or 100 normal control samples.

Total RNA was extracted from the fresh blood samples using Trizol (Takara Dalian, China), and cDNA synthesis was performed using the PrimeScript™ II 1st strand cDNA Synthesis Kit (Takara Dalian, China). PCR reactions were performed to amplify the *PKD*1 cDNA (exon 11-exon 13, ~ 0.3 kb). PCR amplification was performed in a 25 μL total volume containing 2.5 μL of 10× buffer, 1.5 μL of forward primer, 1.5 μL of reverse primer, 0.5 μL of dNTP Mix (10 mM each), 0.2 μL of Taq DNA Polymerase (5 U/μL), 2.0 μL of 25 mM MgCl_2_, 2.0 μL of cDNA and 14.8 μL of deionized water. The following PCR conditions were used incubation at 95 °C for 5 min, 35 cycles of denaturation for 30 s at 95 °C, annealing for 30 s at 60 °C, and extension for 30 s at 72 °C, and a final 5 min extension at 72 °C. The amplified products were used for Sanger sequencing (BigDye® Terminator Cycle Sequencing Kit, ABI3730, America) and TA cloning (CWBIO, Beijing, China). A single clone was then selected for PCR identification and Sanger sequencing. The primer sequences were 5’-AGTGAGGGGGAGCACGTGG-3′ (forward) and 5’-TTCATCCGCTCCACGGTTAC-3′ (reverse). Sequencing reads were analysed with the Chromas software and NCBI BLAST.

**Fig. 3:**
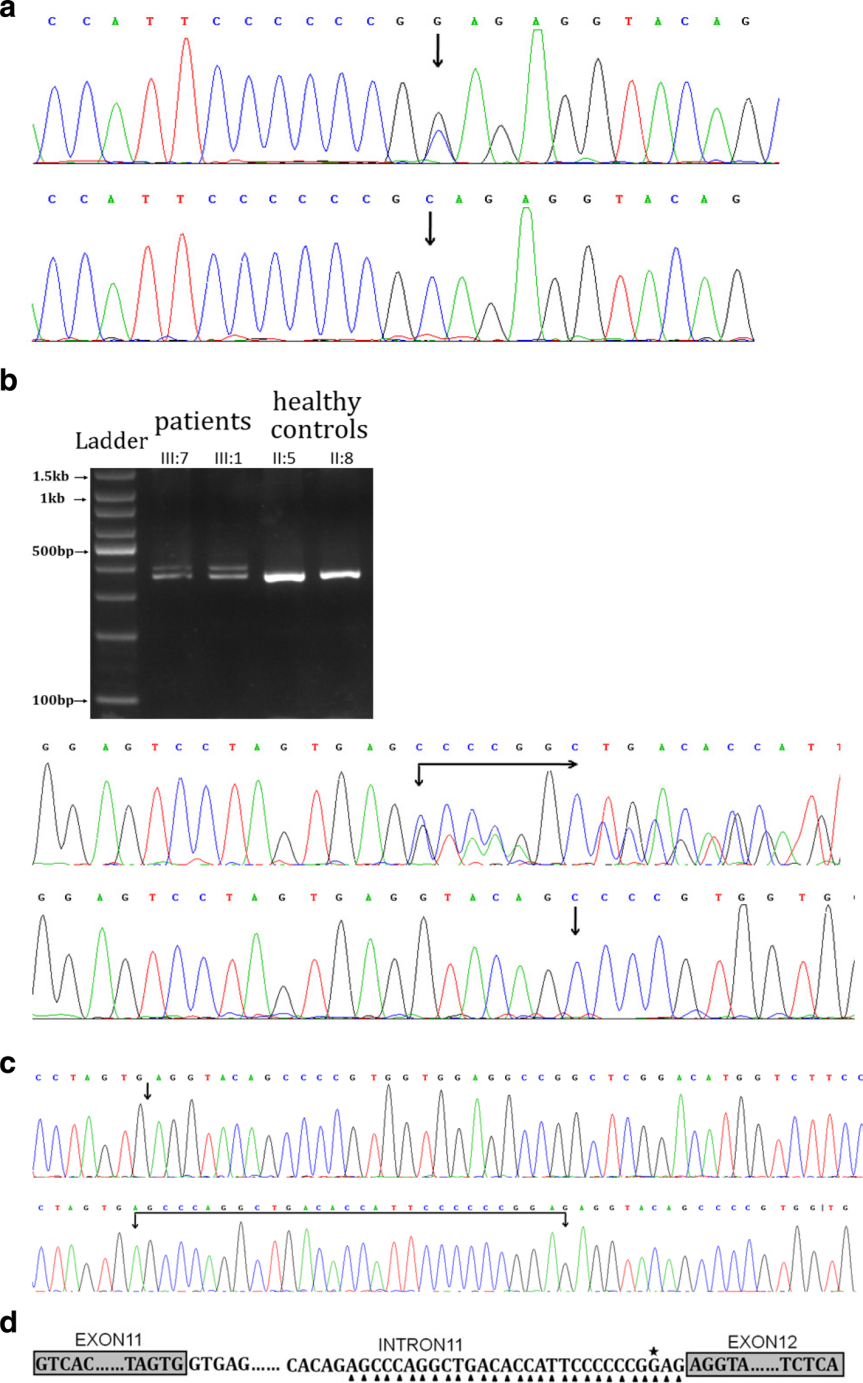
Identification of the novel mutation in the *PKD1* gene. **a** Direct sequencing revealed a heterozygous splice mutation (c.2854-3C > G) in inton11. **a**1: DNA sequence of the patients, **a**2: DNA sequence of the healthy controls, **b** Direct mRNA sequencing, b1: Agarose gel electrophoresis for RT-PCR. b2: mRNA sequence of the patients. b3: mRNA sequence of the healthy controls. **c** The confirmation of transcription by the TA clone. **c**1:The normal patients’ isoforms by TA cloning. **c**2: The mutant patients’ isoforms by TA cloning. **d** The patients’ frameshift mutation. ★-mutant base and ▲-inserted bases. Samples of patients and controls were tested under the same conditions, including equal amounts of sample (peripheral blood and RNA) and experimental conditions

In this typical splicing case, RNA was available for splicing predictions. The sequencing of the reverse transcription–PCR (RT-PCR) products and showed many overlapping peaks from the 3′-end of *PKD*1 gene exon 11, and TA cloning and sequencing confirmed alternative splicing, as 29 bases from the 3′-end of *PKD*1 gene intron 11 were inserted between exon 11 and exon 12, which caused a frameshift mutation (Fig. 3). The frameshift mutation was evaluated using the NCBI ORF Finder (Open Reading Frame Finder) (https://www.ncbi.nlm.nih.gov/orffinder/ ), which showed that of polycystin-1 translation was terminated at the 954th amino acid. The truncated protein is 3349 aa shorter than the wild-type protein (4303 aa).

We classified the variant as “pathogenic” (PVS1+PM2+PP1+PP4) according to the American College of Medical Genetics and Genomics (ACMG) standards and guidelines [11].

## Discussion and conclusions

**Fig. 4:**
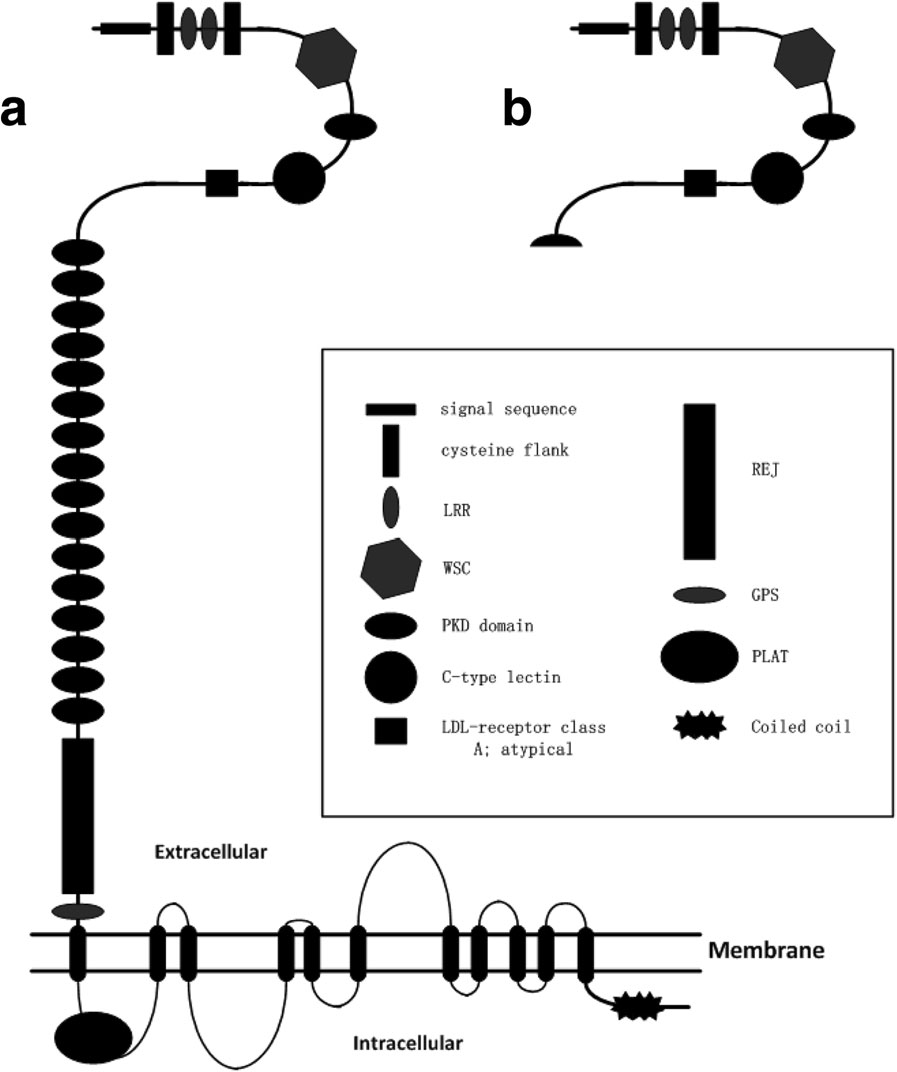
Structure of wild-type and mutant PC1. A-The structure of the wild-type protein. B-The structure of the truncated protein

We reported a novel splice mutation (c.2854-3C > G) in intron 11 of the *PKD1* gene from a family consisting of three patients (II10, III1 and III7) with ADPKD. To further study the effect of *PKD*1 gene transcription caused by this novel splice site mutation, we performed RT-PCR using total RNA from the proband. The cDNA sequence assays showed that 29 bases from the 3′-end of intron 11 in the *PKD*1 gene were inserted between exon 11 and exon 12 in the *PKD*1 cDNA from the proband, which caused a coding region frameshift mutation and led to a 954-amino acid truncated protein (Fig. 4). The truncated protein is part of the extracellular portion of intact polycystin-1 [12, 13], containing the N-terminal initiation region to the first PKD domain and lacking the residual extracellular parts, transmembrane domains and intracellular C-terminal portion. The lack of appropriate C-terminal–mediated signal transduction cascades led to inappropriate regulation of gene transcription and abnormal kidney development [13–15]. The truncated mutant protein cannot function normally to replace intact polycystin-1.

Polycystin-1 is highly expressed in the kidney, brain, liver, pancreas, heart, intestine, etc. and plays an important role in the kidney [12]. Polycystin-1, as an integral part of the cell membrane complex, links the extracellular matrix and the intracellular matrix [16], acting as an adhesion molecule to bind other proteins to form dynamic structures of varying sizes and regulates cell-matrix interactions, motility and signal transduction. In normal renal functioning, polycystin-1 receives information from the extracellular compartment when it binds to ligands via phosphorylation events in its C-terminal region. This transmits intracellular signals to the nucleus to regulate gene transcription appropriately [17]. In abnormal functioning of polycystic-1, the signals cannot be properly transmitted, causing a series of biological changes that lead to pathological changes.

At present, the diagnosis of ADPKD is mainly based on ultrasonography [18, 19], CT scan and MRI imaging [20, 21]. Although regarded to be safe, painless and affordable, these screening and diagnosis methods are not reliable, especially among young patients. Due to similarities in their the clinical phenotypes, there is a risk of confusing the ADPKD with renal cyst syndrome. Therefore, comprehensive analysis is needed, which brings about more challenges for diagnosis with imaging methods. Genomic sequencing technology allowed us to diagnose ADPKD by analysing pathogenic mutations in the *PKD1* and *PKD2* genes [22, 23], as well as to provide health care programmes to ADPKD patients and avoid unnecessary renal damage as early as possible.

A novel *PKD*1 mutation, c.2854-3C > G (also as IVS11–3 C > G), was identified in a Chinese family with ADPKD using NGS and Sanger sequencing. Considering all the elements affecting patients, such as the patient phenotype, mutation location, cDNA frameshift and in-silico prediction analysis, this mutation is considered to play a pathogenic role in the development of ADPKD.

## Abbreviations

aa: Amino acids
ADPKD: Autosomal dominant polycystic kidney disease
CT scan: Computed tomography scan
ESRD: End-stage renal disease
MRI imaging: Magnetic resonance imaging
NGS: Next generation sequencing
ORF Finder: Open Reading Frame Finder
PC1: Polycystin-1
PC2: Polycystin-2
